# Biomaterials Based on Electrospun Chitosan. Relation between Processing Conditions and Mechanical Properties

**DOI:** 10.3390/polym10030257

**Published:** 2018-03-01

**Authors:** Christian Enrique Garcia Garcia, Félix Armando Soltero Martínez, Frédéric Bossard, Marguerite Rinaudo

**Affiliations:** 1 Departamento de Ingeniería Química, Universidad de Guadalajara, Blvd. M. García Barragán #1451, C.P. Guadalajara 44430, Jalisco, Mexico; christian0309@hotmail.com (C.E.G.G.); jfasm@hotmail.com (F.A.S.M.); 2 Institute of Engineering Grenoble Alpes, CNRS, Grenoble INP, LRP, 38000 Grenoble, France; frederic.bossard@univ-grenoble-alpes.fr; 3 Biomaterials Applications, 6 Rue Lesdiguières, 38000 Grenoble, France

**Keywords:** PEO/chitosan blend, swelling, mechanical properties, wet and dried states

## Abstract

In this paper, it is shown that pure chitosan nanofibers and films were prepared with success in 0.5 M acetic acid as solvent using poly (ethylene oxide) (PEO) at different yields, allowing electrospinning of the blends. After processing, a neutralization step of chitosan followed by water washing is performed, preserving the initial morphology of chitosan materials. The influence of the yield in PEO in the blend on the degree of swelling and hydrophilicity of films and nanofibers is demonstrated. Then, the mechanical behavior of blended nanofibers and films used as reference are determined for small stress applied in the linear domain by DMA and by uniaxial traction up to rupture. The dried and wet states are covered for the first time. It is shown that the mechanical properties are increased when electrospinning is performed in the presence of PEO up to a 70/30 chitosan/PEO weight ratio even after PEO extraction. This result can be explained by a better dispersion of the chitosan in the presence of PEO.

## 1. Introduction

Processing of chitosan and chitosan blended with different polymers using electrospinning is often proposed in the literature to produce new biomaterials, especially developed for biomedical applications [1,2,3,4,5,6,7,8,9,10]. The advantage of chitosan is that it is obtained from a natural polymer, chitin, after controlled deacetylation. Chitosan is soluble in aqueous medium in acidic conditions due to –NH_2_ protonation as soon as its degree of acetylation is lower than 0.5 [11]. Then, processing of chitosan is relatively easy, and it can be used under fiber, nanofiber, film, capsule, bead, sponge, gel, powder, tablet based on its insolubility in neutral medium.

Additionally, chitosan is an interesting biodegradable and biocompatible polymer with antibacterial and antifungal properties often described in the literature [12]. In addition, chitosan is stabilized by H–bond network in the solid state providing good mechanical properties under film or fiber materials. The main applications proposed for chitosan in the biomedical domain are: drug delivery, gene delivery vehicle, encapsulation of sensitive drugs, medical textiles, guided bone regeneration, scaffold for nerve tissue regeneration, and wound healing.

In a previous work, the conditions for electrospinning of pure chitosan nanofibers were optimized considering conditions published in the literature [13]. Electrospinning of chitosan has drawn a lot of attention in several scientific studies and a wide range of methods have been used to produce chitosan-based nanofibrous materials in the presence of poly (ethylene oxide) (PEO) as mentioned previously [13,14,15,16,17,18,19]. The optimum condition for a good spinnability and solubility of chitosan was obtained by using 0.5 M acetic acid as solvent and blending with PEO either in solution or in powder. Compatibility of chitosan and PEO was also shown and it was demonstrated that interaction between PEO and chitosan favored the processing. Nanofibers were obtained in the presence of 20 up to 40% (*w*/*w*) PEO yield solutions. In addition, it was confirmed that a chitosan sample with moderate molar mass (*MW* ~ 100,000) is more convenient to control the viscosity of the spinnable solution. In these conditions, nanofibers made of blend PEO/chitosan have average diameters under dried state between 100 and 150 nm [13].

The advantage of electrospinning is that materials are easily produced with high porosity and large surface area which favors the cell development [20,21,22]. Due to its good adhesive characteristic, chitosan is usually blended with other polymers [21]. One reference introduces dibasic sodium phosphate as ionic cross-linker [23]. In these conditions, based on its mechanical characteristics, the chitosan mats are adapted for soft tissue regeneration [23]. The chitosan characteristics are recognized to be used as potential wound dressing for skin lesions due to mechanical properties in the range of that of normal skin [24]. It is very recently that the mechanical properties of these electrospun chitosan-based nanofibers were discussed in literature. It was separately demonstrated that lower degree of acetylation increases the strength of the fibers and decreases the elongation at break for a chitosan/polyvynylalcohol (PVA) (ratio 1/1) electrospun membrane. These authors found a tensile strength at break between 3.5 and 5.2 MPa with 8% and 4% elongation at break when the degrees of acetylation are 16 and 8% respectively [25]. The content of chitosan in the blend chitosan/PVA has also shown to increase the Young modulus and stress at break and to decrease the elongation at break [20]. The values given at 30% of chitosan in the material gives respectively *E* = 201.7 Pa, $\sigma_{break}=$ 4.15 MPa and $\varepsilon_{break}=$ 3.96% [26]. For a chitosan/gelatin system (weight ratio 1/1), these parameters are respectively 48 MPa, 0.478 MPa and 1.3% [27]. Up to now, to our knowledge, no paper concerning the mechanical properties of pure chitosan electrospun nanofibers in dry and wet states are available in the literature. The only published results on wet state concern poly (l-lactide) nanofibers immersed in chitosan and treated with polydopamine at pH = 8.5. Chitosan decreases the stress at break and the elongation. At dried state, in presence of chitosan, the stress at break is around 2 MPa and the elongation is between 40% and 80%. The characteristics of the same samples in the wet state decreases for $\sigma_{break}$ in the range of 0.6–1.0 MPa and increases slowly for $\varepsilon_{break}$ up to 50% to 120% [21]. Nevertheless, the conditions for determination of these important parameters are usually not described.

In the present work, the electrospinning of pure chitosan-based nanofibers was extended to prepare chitosan nanofiber mats and determine their mechanical properties. As reference, films were prepared from the same solutions as used for electrospinning by casting allowing us to compare their physical properties with that of the nanofibers. It will be interesting to test the main differences between unorganized casted and fibrous materials.

For fibers and films production, the two different techniques previously proposed were used: (i) solutions of PEO and solutions of chitosan were mixed and casted in a mold or electrospun; (ii) powdered PEO was added into chitosan solutions [13]. Acetic acid at 0.5 M concentration was adopted as solvent. The change of PEO and chitosan weight ratio and PEO molar mass in the chitosan blends were investigated and related with the electrospun nanofibers and films physical properties under dried and wet conditions.

## 2. Materials and Methods

### 2.1. Materials

Chitosan (CS) sample from Northern cold-water shrimp, *Pandalus borealis* is a gift from Primex Cy (Batch TM4778, code 42010, Siglufjordur, Iceland). Its molecular weight (*MW*) is around 200 kg/mol and its degree of acetylation determined using ^1^H NMR is degree of acetylation (DA) = 0.05. The viscosity of 1% solution is 86 mPa in 1% acetic acid at 25 °C. Poly (ethylene oxide) (PEO) with different molecular weight *MW* (5 × 10^3^ and 1 × 10^3^ kg/mol), acetic acid (≥99.7%), ethanol and K_2_CO_3_ were purchased from Sigma-Aldrich (Saint Quentin Fallavier, France). Deionized water was used as solvent to make up the solutions. All reagents and polymers were used as received without further purification.

### 2.2. Sample Preparation

Chitosan (CS) solutions were prepared separately at 5% (*w*/*v*) in 0.5 M acetic acid. These solutions were prepared at room temperature with slow stirring for 4 days to obtain homogeneous solutions. In the same manner, poly (ethylene oxide) with different *MW* (5 × 10^3^ and 1 × 10^3^ kg/mol) were solubilized at 5% and 3% *w*/*v* in 0.5 M acetic acid on rotating stirrer. Chitosan (CS) solutions were mixed with the solution of PEO at chitosan/PEO weight ratio (in %) of 95/5, 90/10, 80/20, 70/30 and 60/40. Similarly, the same concentration of CS was blended with powdered PEO. The weight ratios were expressed as weight of chitosan or PEO divided by the total polymer weight for each system tested. This addition of powder (PEO) in the solution of chitosan was selected to avoid the dilution of the final chitosan concentration in the polymer blend solution when mixing the two polymers solutions. Viscosities and ionic conductivities of the blended CS/PEO solutions were measured at room temperature.

### 2.3. Chitosan Stabilization

Weighted initial nanofiber mats or films cut in pieces were immersed in alkaline ethanol/water (70/30) mixture dissolving K_2_CO_3_ at pH = 12 to neutralize the chitosan. Further, nanofibers membranes or films were washed for 3 days four times in a day with deionized water until neutral pH to obtain removal of the salt formed from chitosan solutions (potassium acetate), K_2_CO_3_ excess and PEO. At last, the membranes were dried at room temperature for further determination of the swelling capacity or rehydration after a first drying of the materials.

### 2.4. Casting of Chitosan/PEO Films

A certain amount of each of the CS/PEO mixtures showing good spinnability was placed in a Teflon mold of known volume to obtain a uniform polymer film. The probes were stored at room temperature for 3 days until complete evaporation of the solvent.

Different samples of a regular shape were taken from the films obtained for future measurements of their mechanical properties and degree of swelling.

### 2.5. Electrospinning

The prepared solutions were placed in a 5 mL plastic syringe fitted with a 21-gauge stainless steel needle. The syringe pump delivers solutions at specified flow rate vertically (model: KDS Legato 200, KD Scientific, Holliston, MA, USA), and electrospinning is realized with an applied voltage around 20 kV between the electrodes using a homemade dual high voltage power supplier (±30 kV, iseq GmbH, Radeberg, Germany). Then, the nanofibers were recovered on a microstructured collector with a regular pattern supporting an aluminum film used as collector and kept from 10 to 17 cm from the tip of the needle. The flow rates vary from 0.7 to 1.5 mL/h. The experiments were carried out at room temperature in closed Plexiglas^®^ box with relative humidity ranging between 40% and 60%. The produced nanofibers matrices were left in ambient conditions to evaporate excess of acetic acid and water prior to further analyses.

### 2.6. Characterization of Nanofibers

#### 2.6.1. Morphology of the Nanofibers Membranes

The Scanning electron microscopy (SEM) analyses of the samples were performed at CMTC-INP, Grenoble, France. The morphology of electrospun nanofiber membranes including the washed samples were observed with a scanning electron microscope (ultra 55 SEM FEG, Zeiss, Jena, Germany) operated at 3 kV. The nanofibers samples were coated with 10 nm carbon layer prior to SEM imaging. The average fiber diameter (AFD) was calculated by randomly selected diameter of 500 nanofibers from each sample.

#### 2.6.2. Determination of Swelling Capacity

The swelling of the nanofibrous membranes or films were examined in terms of water loss between swollen state in water at neutral pH and final dried weight at room temperature. The wet swollen samples were weighed after blotting with tissue paper to remove excess surface water (*W*_w_). Accordingly, the dried samples were also weighted repeatedly until the mass became constant (*W*_d_). The measurements were carried out three times each. These values correspond to the first swelling. The average data were taken for the determination of swelling ratio *S* using the following equation:

(1) $$S\left(g/g\right)=\left(\frac{W_{w}-W_{d}}{W_{d}}\right)$$

where $W_{w}$ (g) is the weight of the swollen nanofibrous mat or film and $W_{d}$ (g) is the weight of the samples after drying at room temperature.

After drying, rehydration of dried samples was tested under wet form using the same conditions after two days in water at room temperature.

#### 2.6.3. NMR Characterization of Nanofibers

The composition of the nanofibers in chitosan, PEO and acetic acid (or acetate) remaining in the fibers were determined by ^1^H NMR at 80 °C on a Bruker Avance III 400 spectrometer (Billerica, MA, USA). Selected samples were used to analyze the NMR spectrum and exemplify the change in the nanofibers compositions [13]. Nanofiber samples of 7 to 10 mg were dissolved in 1 mL D_2_O in presence of stoichiometric amount of DCl. Analysis of spectra allows the determination of the amount of acetic acid remaining in the dried samples and yield of PEO remaining in the samples after washing in different conditions.

#### 2.6.4. Rheological Behavior

Rheological characteristics were studied using a ARG2 rheometer from TA Instruments (New Castle, DE, USA) with a cone-plate geometry; the cone has a diameter of 25 mm, a 4° angle and a 107 μm gap. The temperature is controlled at 20 °C by a Peltier plate. Steady-state flow experiments were performed in the range of 0.01 to 10 s^−1^.

#### 2.6.5. Ionic Conductivity of Blend Solutions

The conductivity of solution was determined at 20 °C using a Conductimeter sensION+ EC7 from Hach Lange (Loveland, CO, USA) equipped with a titanium electrode Crison 5073. The solvent 0.5 M acetic acid has a conductivity of 1.211 mS/cm.

#### 2.6.6. Mechanical Characterization

The measurements were carried out using ARES-G2 rheometer (TA Instruments, New Castle, DE, USA) equipped with a rectangular geometry, used for axial tension consisting of two axial clamps that hold the material when the force is applied. Samples were taken from the nanofibrous electrospun matrices or films maintaining a length/width ratio around 2.69 (suggested value in the rheometer procedure). The results are expressed as the Stress *σ* = Force applied/section area in Pa. In addition, to compare the samples which have not the same morphology, results of tensile tests are expressed by the reduced force in N/(kg/m^2^) including the mass of the sample divided by its area.

Break tests were performed using the same geometry, starting from a zero-applied force until the material presents a breaking point, with a deformation rate of 0.01 mm/s. The experiments were carried out at constant temperature around 20 °C and a special device was adopted to maintain the relative humidity in the sample environment.

The rheometer also allowed obtaining of the thickness of the samples by measuring the gap between the two plates when they approach film or fiber mat as close as possible until the detector perceives zero axial force during compression. This measurement was repeated with a micrometer (Mitutoyo Digimatic micrometer; −25 mm with precision of 0.001 mm) giving very close values. Both techniques used to determine the thickness are in good agreement with a precision of 1 μm.

The dynamic mechanical analysis Dynamic mechanical analysis (DMA) tests were performed using an initial force applied of 0.02 N with deformations between 0.01% and 0.1% strain imposed by the length/width ratio of the sample. For analysis, the storage moduli *E* observed were normalized taking into consideration the sample weight per unit of surface with the expression:

(2) $$E_{s}\left(\mathrm{Specific}\;\mathrm{modulus}\right)=\frac{\mathrm{Experimental}\;\mathrm{Storage}\;\mathrm{Modulus}\;\left(\mathrm{Pa}\right)}{\frac{\mathrm{Sample}\;\mathrm{weight}\;\left(\mathrm{kg}\right)}{\mathrm{Sample}\;\mathrm{surface}\;\left(m^{2}\right)}\;}$$

## 3. Results and Discussion

To establish the influence of the morphology of chitosan in the materials produced, in a first step, film of chitosan/PEO blends are prepared by casting using the different compositions of the blends. Then, the same solutions were used for electrospinning and production of chitosan nanofibers.

### 3.1. Characterization of Chitosan/PEO Solutions

#### 3.1.1. Viscosity

**Figure 1:**
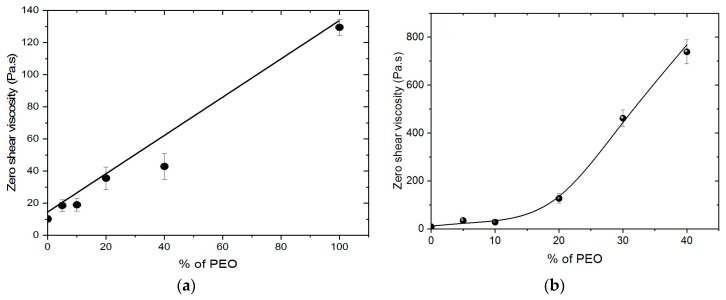
Zero shear viscosity at 20 °C for solutions prepared for films and fibers production as a function of *w*/*w* % of PEO in the blend. *T* = 20 °C. (**a**) By mixing 5% solutions of chitosan and 5% solution PEO in 0.5 M acetic acid; (**b**) by addition of powder PEO in 5% chitosan solution.

The viscosity of the different solutions used to produce films and fibers was determined in steady state conditions at low shear rate. The values at zero shear rate are given in Figure 1a for mixing of the two initial solutions (*w*/*v* 5% chitosan/5% PEO *MW* = 5 × 10^6^) and in Figure 1b for addition of the same PEO in solid state into the 5% chitosan solution.

In Figure 1a, the total polymer concentration remains constant and equal to 5% *w*/*v*. These results indicate good compatibility between the two polymers in the chosen solvent used as mentioned in the literature [13,15,28,29].

As shown in Figure 1b, the viscosity of chitosan increases progressively with increasing PEO ratio. In these conditions, the chitosan concentration remains constant (5% *w*/*v*). Up to the ratio 70/30, the solutions are Newtonian at low shear rate corresponding to electrospinning conditions. These data confirm the compatibility of the chitosan and PEO. The same conclusions were previously obtained with PEO *MW* = 1 × 10^6^ [13].

#### 3.1.2. Conductivity

The conductivity was also determined for the different solutions: all the values vary from 4.5 up to 8.2 mS/cm. At a given chitosan/PEO ratio, the conductivity decreases when the neutral PEO weight fraction increases whatever the blend conditions (mixture of solutions or PEO powder added in chitosan solution). However, the conductivity of mixed solutions remains slightly lower than when powder is added in chitosan solution due to a larger content of chitosan in this case (5% weight concentration). A small increase of conductivity with decrease of the viscosity is also observed when conductivity of chitosan/PEO blend with 5% *MW* = 1 × 10^6^ is compared with that of blend with 5% *MW* = 5 × 10^6^ at the same chitosan/PEO ratio.

### 3.2. Casting and Film Characterization

#### 3.2.1. Degree of Swelling of Films

After casting and dying in atmospheric conditions, the chitosan in the blended films is neutralized in non-solvent basic conditions (ethanol/water 70/30 *V/V* containing CO_3_K_2_) and washed with fresh water until neutrality. The chitosan becomes insoluble at pH > 6.5 and PEO is solubilized in water. To control this step, the dried weight of the sample was determined in the same time as the degree of swelling (using the wet weight after washing step). All the data are given in Table 1. Taking these results into consideration, after drying, the samples are stabilized in water to determine the degree of rehydration of the chitosan materials, the hydrophilic character being important for biological application.

From Table 1, it is clearly shown that the dried weight after rehydration remains the same as after the first step of drying indicating extraction of PEO. Nevertheless, the values remain slightly higher than the predicted value based on the initial weight fraction of chitosan especially when the PEO is added in the powder form. This is probably due to localization of PEO in the chitosan compact matrix. A small PEO fraction within the film may be not accessible for the washing process. The final composition of the sample was examined by ^1^H NMR as previously described [13]. On films prepared at 80/20 chitosan/PEO ratio, it is confirmed that a small amount of PEO was not extracted in the process adopted especially when PEO is added under powder form.

**Figure 2:**
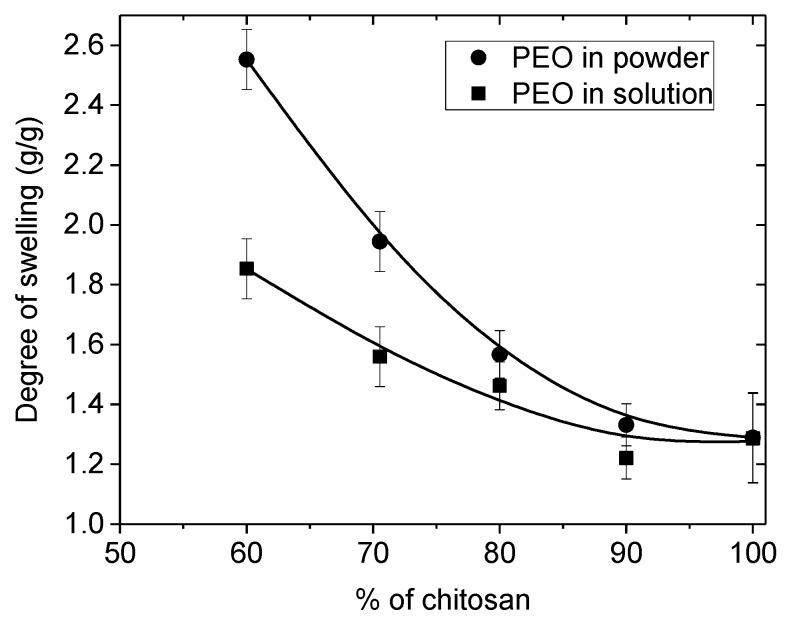
Influence of the initial PEO content in rehydration of chitosan films as a function of *w*/*w* % of chitosan in the blend (● powder form; ■ solution 5%, *MW* = 1 × 10^6^).

The degree of swelling of the films is directly related with the composition of the blend as shown in Figure 2. The larger is the initial PEO yield, after extraction of the PEO, the larger is the swelling degree in water i.e., also of the specific area. In addition, it is shown that the hydrophilic character reflected by the degree of swelling remains high even after complete drying of the PEO extracted films. Then, the role of the yield in PEO on the properties of the material prepared remains important.

It may be added also that when PEO is added under powder form, the chitosan concentration is larger in the electrospun blend than in case of mixed solutions. After extraction of PEO and drying the pure chitosan film swelling is larger in relation to a larger porosity.

#### 3.2.2. Mechanical Characteristics of Films

**Figure 3:**
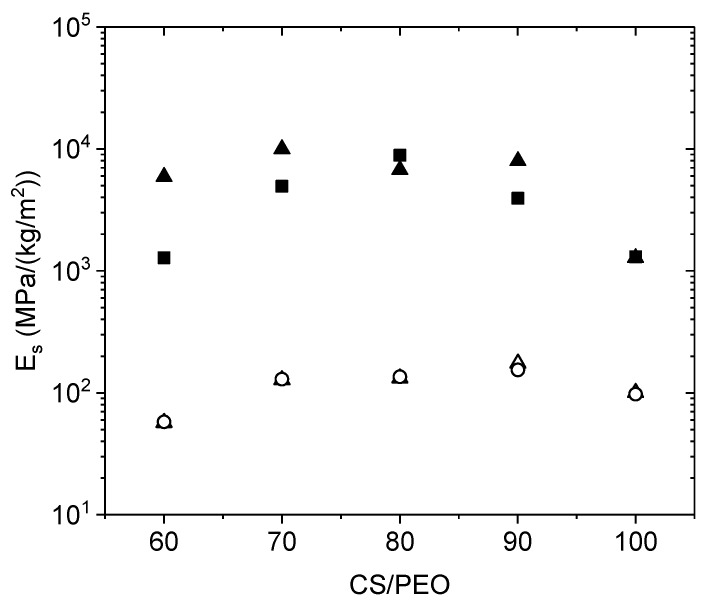
Specific modulus *E_s_* expressed in MPa/(kg/m^2^) obtained by DMA on initial blended film for powdered PEO added in 5% chitosan solution (dried state ▲, ■) and after PEO (*MW* = 1 × 10^6^) extraction, after drying and rehydration (wet state ∆, ○). Standard deviation is lower than 10%.

Firstly, DMA was performed under a low force (0.02 N) in the linear domain giving the elastic modulus *E* expressed in Pa. To compare results from one sample to another, moduli were normalized by the mass per unit area of each samples and expressed as *E_s_* in Pa/(kg/m^2^). Mechanical tests on membranes based on chitosan/powder PEO were performed both on the same sample just after the electrospinning process and after PEO extraction (Figure 3, Table 2).

The modulus of the film in the initial dried state is reinforced by the presence of PEO, nevertheless for biological application the properties under the chitosan insoluble form in aqueous medium must be determined. For that purpose, the films were neutralized and the PEO was extracted to perform the test under the swollen state at equilibrium in aqueous medium. Even after PEO extraction, the elastic modulus is reinforced when the chitosan was blended with PEO at least up to 70/30 chitosan/PEO ratio. Considering the data given in Table 2, it is shown that there is a slight influence of the conditions of PEO mixing (powder or solution) but it appears that the stronger performance in the wet state are obtained with solution of the higher molar mass PEO.

An interesting characteristic is the ratio between initial modulus in the dried state and the modulus in the wet state after PEO extraction. This ratio is very large when PEO is added in the powder form and it decreases when the content in PEO decreases. It indicates a larger porosity of the films formed in presence of PEO. The ratio remains lower when solutions are mixed corresponding to a larger homogeneity of the materials as well as a lower degree of swelling (see Figure 2).

**Figure 4:**
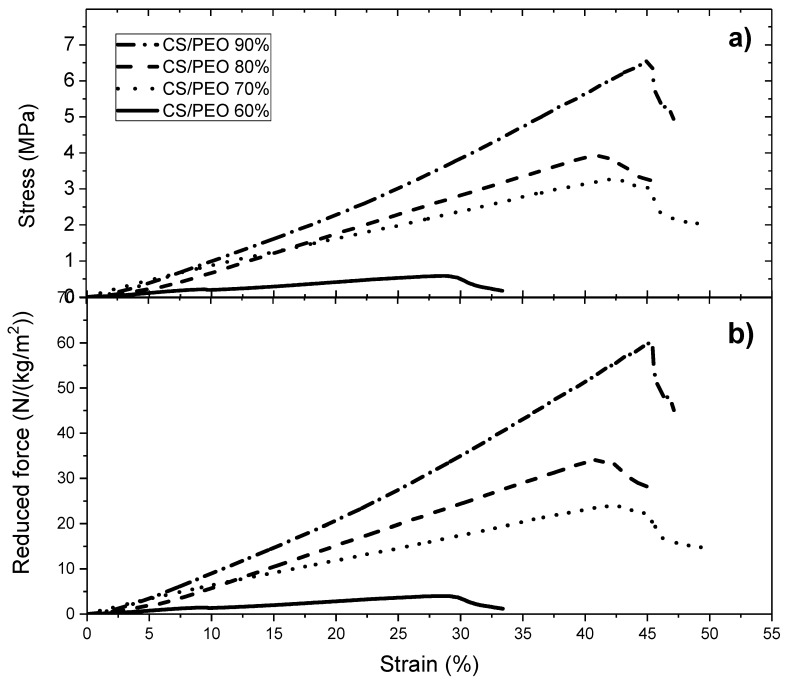
(**a**) Stress under uniaxial traction *σ* (expressed in MPa) and (**b**) Reduced force (expressed in N/(kg/m^2^)) on films under rehydrated form (wet state) after chitosan neutralization, PEO (under powder form) extraction and drying. PEO *MW* = 1 × 10^6^.

Tests of uniaxial traction stress were performed on the wet films after PEO extraction. The blending with powder and solution PEO are compared in Figure 4a. To be able to compare all the experimental results and especially because the different materials prepared are porous, the performances are expressed by the force in Newton (N) reduced by the surfacic mass (kg/m^2^) are represented in Figure 4b.

From Figure 4b, it is clear that the mechanical properties of the films in the wet state are directly related to the chitosan content in the initial casted film. The density of chitosan is larger when the yield of chitosan in the blend is larger.

Down to a ratio 60/40 chitosan/PEO, the strain at break remains nearly constant around 40%. It must be pointed out that the wet films have a high stress at beak (*σ* ~ 7 MPa) when prepared at high chitosan content (Table 3). This value is clearly higher than values given in the literature for different blends under dried state.

From this table, it is shown that the stress at break is increased when the films are prepared in the presence PEO (up to 70% CS/PEO) as well as the degree of extension and that there is no important difference in dependence of the conditions of PEO addition (powder or solution). It is suggested that mixing chitosan and PEO allows better dispersion of chitosan molecules and more homogeneous films with stronger H bond stabilization.

### 3.3. Electrospinning of Blends

#### 3.3.1. Conditions of Electrospinning

Different experimental conditions were explored to optimize the production of nanofibers at high yield in chitosan using two PEO with different molar masses. The average parameters determined to get fibers in absence of spray or beads are given in Table 4.

**Figure 5:**
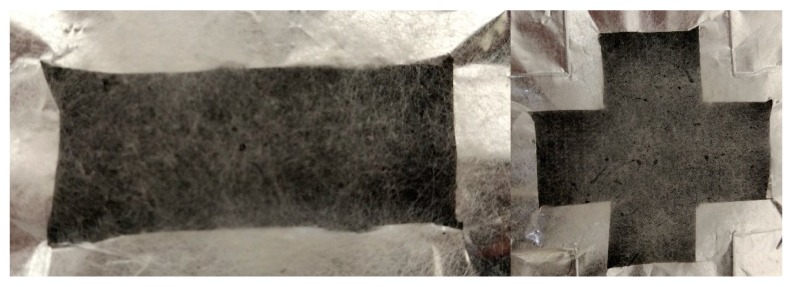
Experimental devices for nanofibers production.

In our electrospinning process, the collector was designed permitting to recover the fibers matrix from a metal plate avoiding sticking on the support (Figure 5). Aluminum foils cut in cross or rectangle have been chosen as support to remove the mat after processing as shown in Figure 5. In these conditions, the probes for mechanical tests are easy to take out.

Considering the experimental conditions given in Table 4, it must be noticed that they are different, especially considering the flow rates, compared with our previous work even if the solvent is the same as well as one of the PEO (*MW* = 1 × 10^6^) [13]. In this previous work, the flow rates varied between 20 and 100 μL/h and the viscosities of systems were much lower than in the present study. It is due to the higher molar mass of the chitosan used in the present work as well as PEO molar mass of 5 × 10^6^. It is important to point out the importance of the molar mass of the chitosan used as well probably of its degree of acetylation which controls the solubility. PEO with *MW* = 5 × 10^6^ was preferred to get stronger fibers in the following part of the work.

#### 3.3.2. Degree of Swelling of the Nanofibers

From the yield in polymer remaining after extraction of PEO, Table 5 indicates that PEO is nearly totally extracted. ^1^H analysis confirmed this information by small signal located at 3.8 ppm corresponding to the superposition of one H of chitosan and –CH_2_– of PEO left.

Due to porosity of the mat of fibers, the water content corresponds to the swelling of the fibers but also to water included in the porous materials between hydrophilic fibers. Then, the values are larger than values obtained on films prepared from the same solutions. In addition, it is found that the dried weight fraction obtained compared to the initial chitosan dried weight fraction indicates nearly complete PEO extraction as confirmed by NMR experiments. Then, these data demonstrate that accessibility for extraction is much better under fiber morphology compared to the films (compare with Table 1).

**Figure 6:**
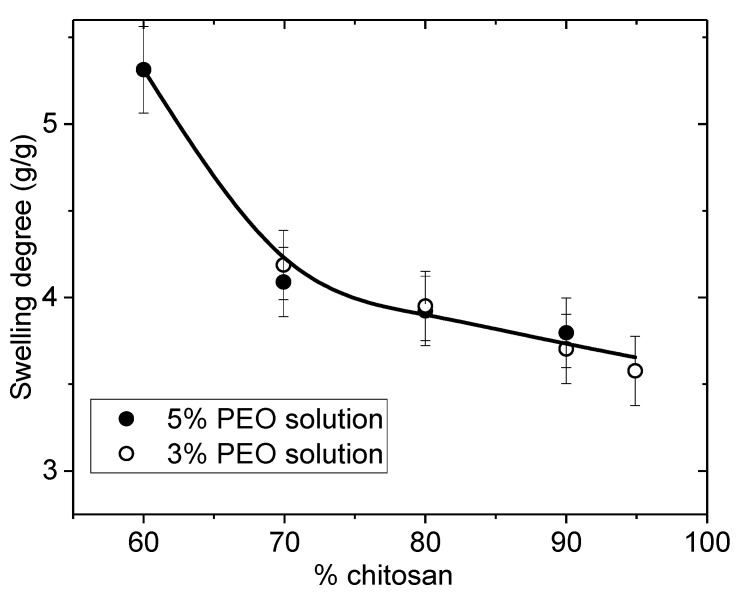
Degree of swelling of nanofiber mats after extraction of PEO (*MW* = 5 × 10^6^) and rehydration as a function of the initial chitosan content *w*/*w* % in the initial blend.

The influence of the PEO content in the blend controls the degree of rehydration which increases when PEO content increases as represented in Figure 6. The values are nearly the same for the two PEO concentrations used during electrospinning.

The swelling of nanofibers in the presence of PEO powder in the blend was found larger than when using solutions as observed previously with films. The nanofibers are better dispersed when blended with PEO solutions giving a more homogeneous network. The hydration is larger than that of films due to the lower chitosan density of the nanofiber mats compared with the casted films. This result indicates a larger porosity and larger accessible surface of the fiber mat compared with film. This point should be important for biological applications and especially cell developments.

#### 3.3.3. Mechanical Characterization of Nanofibers

The characterization of the mechanical properties of the matrices obtained by electrospinning and their relationship with those of the casted non-structured materials is the fundamental part of this research work. Therefore, different stages in the measurement of properties were carried out. For this purpose, a sequence of dynamic mechanical analysis (DMA) was chosen where small deformations are applied to the material without affecting its structure, nor causing significant elongation, being able to recover the sample for subsequent treatments (Table 6). This sequence ends with material break tests using uniaxial deformation under the wet state.

From this table, it comes that the ratio between initial modulus and swollen wet modulus are much lower than for films (compare with Table 2). This indicates a larger stability of the fibrillary network compared with films. In addition, modulus in the hydrated form remains higher when PEO was initially introduced in 5% solution even if in the initial dried state, it is the opposite due to the degree of distribution of the fibers and larger interfaces but lower cross section. It must be pointed that the *E_s_* values in the swollen state are much higher than on film for the same composition even if the chitosan density is lower in the mat.

The data for uniaxial traction up to break are analyzed in Table 7.

From these data, it is shown that in the initial dried state, the performance of the material is better when the blend is prepared by mixing the solutions even if the yield in chitosan during preparation is lower in this case (due to mixing of the two solutions) compared to addition of solid PEO. The higher is the chitosan content the higher is $\sigma_{Break}$ and lower is the elongation ($\varepsilon_{Break}$ %). From these data, it is concluded that normalized *σ_break_* values are slightly larger than for the corresponding chitosan/PEO composition obtained with the films even if the density of the mat is lower (Table 3).

**Figure 7:**
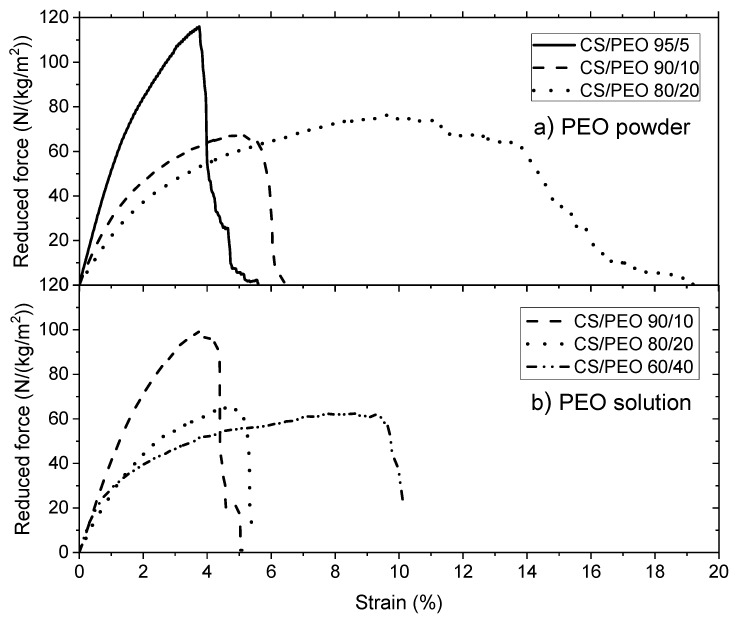
Uniaxial extension for chitosan/PEO nanofibers blends in the initial state. (**a**) PEO *MW* = 5 × 10^6^ added under powder form; (**b**) PEO mixed in solution from *MW* = 5 × 10^6^ at 3%.

An example of mechanical properties of chitosan/PEO nanofibers blends is given in Figure 7a,b, in which the mechanical behavior is compared when PEO is added under powder form and in solution respectively. The elongation at break increases with increasing the ratio of PEO. These results point out that PEO interacts with chitosan as shown previously [13]. Then, PEO plays the role of a plasticizer and the material becomes less brittle.

**Figure 8:**
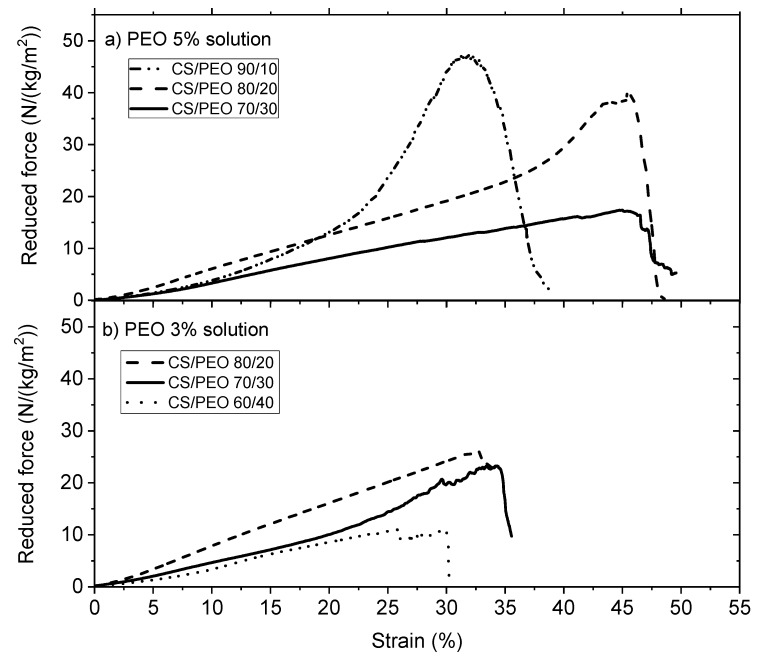
Uniaxial extension for chitosan nanofibers in the wet state after PEO extraction (**a**) PEO mixed in solution from *MW* = 5 × 10^6^ at 5% (**b**) PEO mixed in solution from PEO 5 × 10^6^ solution at 3%.

Then, the performances were compared under wet state after PEO extraction and rehydration. Few results are given in Figure 8 in which the influence of the PEO concentration is tested.

From Figure 8, it is clearly shown that the mechanical behavior of the chitosan nanofibers is stronger when the yield of chitosan increases with a relatively high elongation around 30% to 35% under wet state. In the presence of 5% PEO solution having a higher viscosity but also a higher chitosan concentration for the same chitosan/PEO ratio, the stress at break is higher probably due to a lower dispersion of chitosan fibers with higher degree of packing of the chitosan molecules causing H–bonding in the intermediate drying of the materials.

It must be pointed out that the influence of PEO in the blend on the reinforcement of the mechanical properties is lower than for the films and the values of $\sigma_{Break}$ are of the same order of magnitude with a lower density of chitosan (compare Table 1 and Table 5).

#### 3.3.4. Morphology of the Nanofibers

Different states of the fibers mats were examined by scanning electron microscopy to point out the role of the blend composition and the role of PEO extraction on the morphology of the fibers.

**Figure 9:**
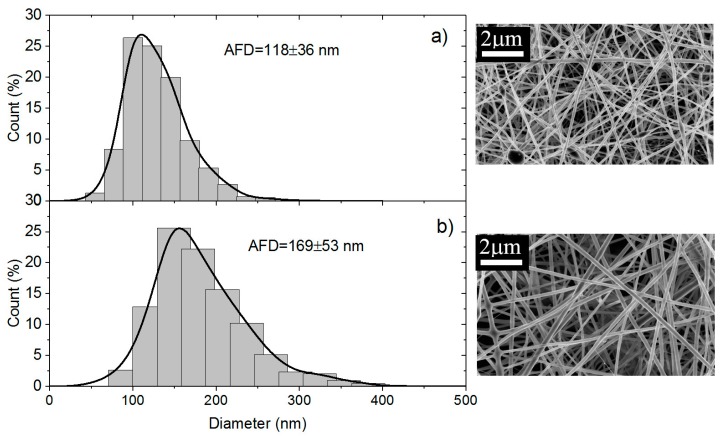
Diameter distribution of chitosan/PEO fibers expressed as a function of the count of fibers in % for (**a**) PEO *MW* = 10^6^ at the concentration of 5% mixed with chitosan solution and (**b**) PEO *MW* = 10^6^ added in powder form.

Some examples are given in the following pictures in which nanofibers produced at 70% and 95% chitosan are compared (Figure 9). From their analysis, it is shown that the average diameters of 70% chitosan fibers produced with PEO powder are larger than when 5% solution is used with PEO *MW* = 1 × 10^6^ (Figure 9a,b).

**Figure 10:**
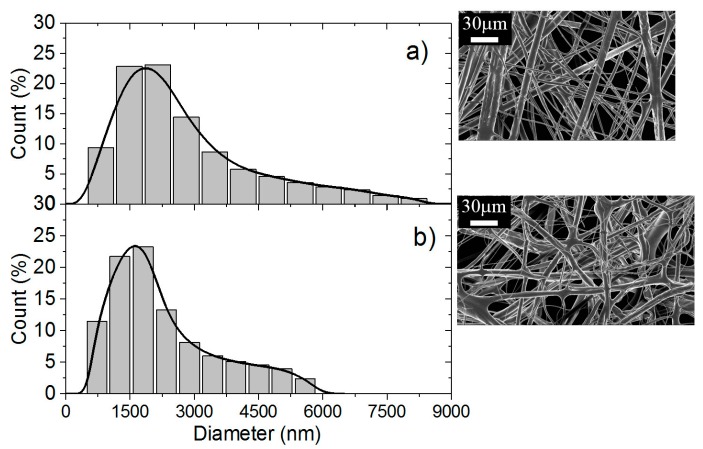
Diameter distribution of chitosan/PEO fibers expressed as a function of the count of fibers in % for PEO *MW* = 5 × 10^6^ at the concentration of (**a**) 5% and (**b**) 3%.

In addition, the diameters of the fibers are lower for 70% chitosan when PEO is added as 5% PEO solution (*MW* = 1 × 10^6^) (Figure 9a) compared with PEO (*MW* = 5 × 10^6^) (see Figure 10a). This influence of *MW* was previously shown for cellulose acetate and poly(vinyl chloride) nanofibers [30]. SEM indicates that fibers are thinner with 3% solution than with 5% with also a broader distribution causing lower mechanical properties (Figure 10a,b). These results are directly related with the viscosity of the electrospun systems and consequently with the flow rate necessary to optimize the process.

**Figure 11:**
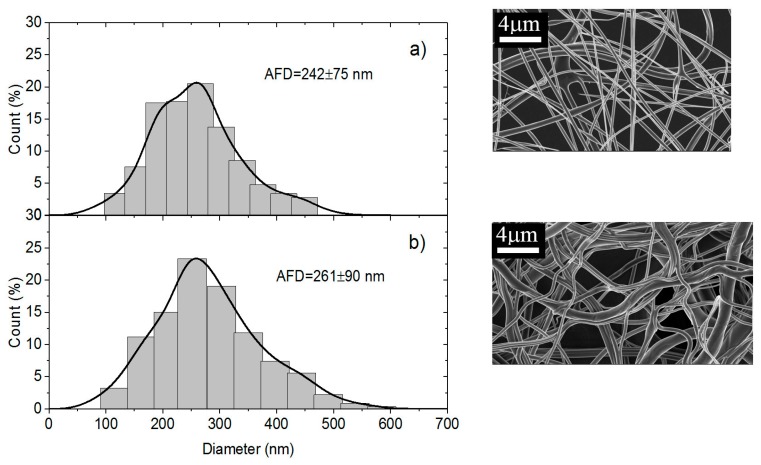
Diameter distribution of chitosan/PEO fibers expressed as a function of the count of fibers in % for PEO *MW* = 5 × 10^6^ at the concentration of 5% (**a**) before washing and (**b**) after washing.

Concerning the influence of the chitosan/PEO ratio, using PEO *MW* = 5 × 10^6^ at 5% mixed with 5% chitosan solution, the fiber diameters for 70/30 are 10 times higher than for the 95/5 ratio (Figure 10a and Figure 11a). Such larger diameter distribution is essentially due to much higher viscosity of the 70/30 system (see Figure 1a,b), all the parameters for electrospinning being equivalent.

The last parameter tested is the washing step as shown in Figure 11.

Comparison of Figure 11a,b indicates that washing does not modify the fiber morphology. Nevertheless, the distribution of diameters is a little broader than that obtained for 70/30 in presence of PEO *MW* = 1 × 10^6^ but the use of PEO *MW* = 5 × 10^6^ was necessary to get convenient conditions for electrospinning even if the viscosity was larger for the PEO solution.

## 4. Conclusions

In this paper, the influence of the structure of materials based on chitosan was examined. Firstly, films were casted from PEO/chitosan blends in different conditions. It was clearly shown that the degrees of swelling and mechanical properties of these chitosan films are controlled by the content and conditions of PEO addition even after extraction of PEO. The degree of swelling increases when PEO content increased up to 60% in the blend. In the same time, the specific modulus *E_s_* from DMA increases in the initial dried state as well as in the wet state after PEO extraction corresponding to a better dispersion of chitosan and stronger inter-chain interactions (involving H-bond stabilization). Such chitosan films prepared with 90% chitosan in the initial blend with PEO powder (*MW* = 1 × 10^6^) and after PEO extraction have stress at beak around 6.6 MPa or 60 N/(kg/m^2)^ with an elongation at beak larger than 40% in the wet state. These results confirm the good film forming character of chitosan recognized for a long time. The films are less brittle when casted from the blend due to their more porous and hydrophilic structure.

Secondly, using the same solutions, electrospinning was performed in experimental conditions optimized to get good nanofibers which were tested in the initial state but also after PEO extraction in the wet state. This study allows discussion of the advantage of preparing chitosan nanofibers among unstructured films. The PEO extraction is more efficient under fiber morphology as shown by NMR. The density decreases and the degree of hydration of nanofiber mats increases much when processed in presence of PEO compared with film. The ratio between *E_s_* values in the initial composite state and in the wet state on pure chitosan remains lower than on films corresponding to a larger stability of the network formed. In the same time, the specific modulus *E_s_* is much larger on the mats in the wet state than under film structure even if the density is lower. This is attributed to the larger porosity and higher dispersion and higher degree of connection between the chitosan chains. This aspect is certainly interesting for cell development. At 90% chitosan blended with 5% PEO solution (*MW* = 5 × 10^6^), after extraction of PEO in the wet state, the stress at break is around 1.2 MPa or 47 N/(Kg/m^2)^ and 32% elongation at break which are of the same order of magnitude than for films even with a lower density of fibrous material. One advantage of the nanofibers is the large porosity of membranes as well as its large specific surface allowing a possible high surface adsorption.

To conclude, for the first time, chitosan nanofibers mats are prepared and characterized completely for their structural and mechanical characteristics in the dried and wet state allowing the use of these materials for biological applications in the near future. In addition, it must be remembered that these fibers are stable in aqueous medium at pH higher than chitosan *pK*_0_ for years.

## Figures and Tables

**Table 1:** Degree of swelling of blended film and composition after chitosan neutralization.

Systems	Chitosan Weight Ratio %	Film Thickness (mm)	Density (g/cm^3^)	Initial Swelling Degree after PEO Extraction (g/g)	Remaining Polymer Fraction after First Neutralization	Swelling Degree after Rehydration (g/g)	Remaining Polymer Fraction after Rehydration	Theoretical Chitosan Fraction in the Material
	100	0.108	0.781	1.893	0.99	1.291	0.98	1.0
**Powdered PEO added**	90	0.092	0.767	1.852	0.96	1.372	0.95	0.9
	80	0.078	0.923	1.786	0.93	1.589	0.92	0.8
	70	0.085	0.978	2.439	0.86	1.966	0.85	0.7
	60	0.124	1.041	2.954	0.72	2.544	0.70	0.6
**Blend of CS and PEO solutions**	90	0.079	0.967	1.410	0.95	1.222	0.94	0.9
	80	0.083	0.817	1.767	0.90	1.466	0.89	0.8
	70	0.078	0.757	2.809	0.69	1.564	0.68	0.7
	60	0.068	1.004	1.939	0.64	1.860	0.64	0.6

**Table 2:** Experimental values of *E_s_* obtained in DMA on initial blended films and after extraction of PEO with different *MW* and concentrations being added in powder or solution.

Systems	$\frac{CS}{PEO}$	*E_s_* $\left(\frac{MPA}{kg/m^{2}}\right)$	*E_s_* $\left(\frac{MPA}{kg/m^{2}}\right)$	$\frac{E_{initial}}{E_{swollen}}$	$\frac{CS}{PEO}$	*E_s_* $\left(\frac{MPA}{kg/m^{2}}\right)$	*E_s_* $\left(\frac{MPA}{kg/m^{2}}\right)$	$\frac{E_{initial}}{E_{swollen}}$
		Initial State	Wet State after Rehydration			Initial State	Wet State after Rehydration	
	PEO *MW* = 1 × 10^6^ g/mol				PEO *MW* = 5 × 10^6^ g/mol			
PEO powder	60	5970	55	108				
	70	9970	126	79				
	80	6760	134	50.6	80	3830	166	22.99
	90	7820	176	44.5	90	2350	183	12.84
	100	1300	98	13.3	95	1590	201	7.92
5% PEO solution	60	1310	-	-				
	70	5080	-	-	80	3430	306	11.19
	80	8930	142	62.8	90	3370	294	11.46
	90	3990	166	24.0	95	2230	244	9.15
3% PEO solution					70	1070	170	6.26
					80	1320	260	4.99
					90	1010	240	4.19

**Table 3:** Experimental data obtained in uniaxial tensile test on blended films after PEO extraction (PEO 1 × 10^6^) and drying under wet state.

CS/PEO Powder	$\sigma_{Break}$		$\varepsilon_{Break}$	CS/5%PEO Solution	$\sigma_{Break}$		$\varepsilon_{Break}$
%	MPa	$\frac{N}{kg/m^{2}}$	%	%	MPa	$\frac{N}{kg/m^{2}}$	%
100	1.41	14	33.2	100	1.41	14	33.2
90	6.57	60	45.1	90	5.12	43	44.5
80	3.95	34	41.2	80	3.91	38	19.4
70	3.28	24	42.6				
60	0.66	4	29.2				

**Table 4:** Experimental conditions for electrospinning on the different systems studied

**PEO *MW* = 1 × 10^6^**				
**Powdered PEO Added into the Chitosan Solution**				
**CS/PEO**	**Pump Rate (mL/h)**	**Tip to Collector Distance (cm)**	**Applied Voltage (kV)**	**Electrospun Products**
70/30	0.2–0.4 *	11 *–12	24–27 *	Fibers
80/20	0.22 *–0.25	13	25–27 *	Fibers
**Mixture of the Polymer Solutions**				
60/40	0.05–0.2 *	14	22–25 *	Fibers
70/30	0.09–0.1 *	14	27	Fibers
**PEO *MW* = 5 × 10^6^**				
**Powdered PEO Added into the Chitosan Solution**				
80/20	1.0–1.15	13–15	21–25	Fibers
90/10	1–1.1	12–15	20–24	Fibers
95/5	0.7–1.5	14 *–17	21–24 *	Fibers, few beads
**Mixture of the Polymer Solutions (5%)**				
70/30	1.4–1.5	15	24–27	Fibers
80/20	0.6 *–1.0	15	21–24 *	Fibers
90/10	0.65–0.7	15	20	Fibers
95/5	1.2	15	22	Fibers, few beads
**Mixture of the Polymer Solutions (3%)**				
60/40	0.7 *–1.4	15	20–24	Fibers, few beads
70/30	0.8 *–1.3	14 *–17	21–22 *	Fibers
80/20	0.7–0.8 *	13 *–14	20–22 *	Fibers
90/10	0.7 *–0.9	14 *–16	21 *–24	Few beads, fibers

* Indicates the optimum conditions.

**Table 5:** Degree of rehydration of nanofibers after neutralization, extraction of PEO (*MW* = 5 × 10^6^) and drying.

Systems	Fiber Mat Thickness (mm)	Chitosan Weight Concentration Ratio	Density (g/cm^3^)	Rehydration Degree (g/g)	Remaining Polymer Fraction after Rehydration	Theoretical Chitosan Fraction in the Material
Powdered PEO added	0.16	95	0.117	3.13	0.93	0.95
	0.156	90	0.132	4.29	0.91	0.90
	0.127	80	0.141	4.50	0.84	0.80
Blend of CS and 5% PEO solution	0.128	95	0.173	3.58	0.90	0.95
	0.122	90	0.163	3.71	0.88	0.90
	0.102	80	0.200	3.95	0.84	0.80
	0.115	70	0.164	4.19	0.73	0.70
Blend of CS and 3% PEO solution	0.115	90	0.169	3.80	0.95	0.90
	0.120	80	0.192	3.93	0.83	0.80
	0.112	70	0.176	4.09	0.73	0.70
	0.114	60	0.167	5.33	0.65	0.60

**Table 6:** Experimental values of *E_s_* obtained in DMA on initial blended fibers and after PEO extraction (*MW* = 5 × 10^6^) used in powder form and in 5% and 3% solutions under swollen state after drying and rehydration.

Systems		*E_s_* $\left(\frac{MPA}{kg/m^{2}}\right)$ Initial State	*E_s_* $\left(\frac{MPA}{kg/m^{2}}\right)$ Wet State after Rehydration	$\frac{E_{initial}}{E_{swollen}}$
CS/PEO powder	80	1650	466	3.53
	90	1460	434	3.36
CS (5%)/PEO solution	70	939	421	2.23
	80	1490	652	2.28
	90	1370	602	2.28
CS (3%)/PEO solution	60	2430	432	5.63
	70	2760	510	5.41
	80	2580	546	4.73

**Table 7:** The uniaxial stress/strain properties on nanofibers mat under hydrated form after neutralization and PEO extraction, drying and rehydration. (*MW* = 5 × 10^6^).

[CS]	$\sigma_{Break}$		$\varepsilon_{Break}$	[CS]	$\sigma_{Break}$		$\varepsilon_{Break}$	[CS]	$\sigma_{Break}$		$\varepsilon_{Break}$
%	MPa	$\frac{N}{kg/m^{2}}$	%	%	MPa	$\frac{N}{kg/m^{2}}$	%	%	MPa	$\frac{N}{kg/m^{2}}$	%
*Powder PEO*				*5% Solution*				*3% Solution*			
								60	0.4	11	30.4
				70	0.42	18	44.9	70	0.64	23	34.5
80	0.83	34	53.8	80	1.43	40	46.0	80	0.72	26	32.8
90	0.61	23	41.2	90	1.19	47	31.9				
